# MR Imaging in Real Time Guiding of Therapies in Prostate Cancer

**DOI:** 10.3390/life12020302

**Published:** 2022-02-17

**Authors:** Yvonne Wimper, Jurgen J. Fütterer, Joyce G. R. Bomers

**Affiliations:** Department of Medical Imaging, Radboud University Medical Centre, Geert Grooteplein 10, 6525 GA Nijmegen, The Netherlands; Jurgen.Futterer@radboudumc.nl (J.J.F.); Joyce.Bomers@radboudumc.nl (J.G.R.B.)

**Keywords:** prostate cancer, focal therapy, minimal-invasive treatments, MRI-guided therapies, cryoablation, focal laser ablation, high intensity focused ultrasound, transurethral ultrasound ablation

## Abstract

Magnetic resonance imaging (MRI)-guided therapy for prostate cancer (PCa) aims to reduce the treatment-associated comorbidity of existing radical treatment, including radical prostatectomy and radiotherapy. Although active surveillance has been used as a conservative method to reduce overtreatment, there is a growing demand for less morbidity and personalized (focal) treatment. The development of multiparametric MRI was of real importance in improving the detection, localization and staging of PCa. Moreover, MRI has been useful for lesion targeting within the prostate, as it is used in the guidance of prostate biopsies, by means of cognitive registration, MRI-ultrasound fusion guidance or direct in-bore MRI-guidance. With regard to PCa therapies, MRI is used for precise probe placement into the lesion and to accurately monitor the treatment in real-time. Moreover, advances in MR-compatible thermal ablation allow for noninvasive real-time temperature mapping during treatment. In this review, we present an overview of the current status of MRI-guided therapies in PCa, focusing on cryoablation, focal laser ablation, high intensity focused ultrasound and transurethral ultrasound ablation. We explain the important role of MRI in the evaluation of the completeness of the ablation and during follow-up. Finally, we will discuss the challenges and future development inherent to these new technologies.

## 1. Introduction

Since the discovery of prostate-specific antigen (PSA) in the 1970s the disease management of prostate cancer (PCa) has shifted tremendously [1]. Current practices allow for early detection of PCa, whereas historically the diagnosis was made when metastases were present. The widespread use of PSA in the clinical setting made it possible to increase the detection of PCa and it was followed by a reduction in PCa deaths, but unfortunately also with an increased treatment-associated morbidity [2,3].

Based on histopathological verification, acquired by prostate biopsies, a risk stratification can be made into low- (Gleason score ≤6/International Society of Urological Pathology (ISUP) Grade Group 1), intermediate- (Gleason score 7/ISUP Grade Group 2/3) and high-risk PCa (Gleason score >7/ISUP Grade Group 4/5). The Gleason score is formed by the sum of the two most prevalent differentiation patterns varying between 1 and 5. Current active treatment in localized low-, intermediate- and high-risk PCa consists of whole gland treatment, i.e., radical prostatectomy (RP) and radiotherapy (including external beam radiotherapy (EBRT) and brachytherapy). These therapies are associated with a high risk of developing incontinence (16%) and erectile dysfunction (34%) [4,5], and a substantial risk of developing second malignancies of the bladder, colon and rectum (adjusted hazard ratio of 1.67, 1.33–1.79 and 1.64–1.79), urethral stricture (2.2%) and bowel dysfunction [6,7,8,9]. To reduce overtreatment, a conservative management with active surveillance (AS) has been conceived for patients with localized low-risk PCa (Gleason score 3 + 3 = 6/ISUP Grade Group 1), because low-risk PCa grows very slowly and has a low risk of metastasis. AS involves monitoring the PCa and only proceeding to treatment when there is progression of the tumor. Despite the fact that AS seems to offer a great advancement, only about forty percent of the patients with a low-risk PCa choose AS [10] and one-third of patients on AS will still require an active treatment in the future [11]. It is not surprising that the urological and radiological community began to explore opportunities to reduce treatment-associated morbidity.

There is a great need for individualized therapy in order to find a balance between the potential risk of disease progression in patients on AS and overtreatment due to radical therapy. Individualized therapy, based on adequate patient selection and precise disease localization for patients with localized PCa, could provide the answer for increasing treatment success. Several types of (image-guided) minimal-invasive treatment options have been developed in the last decades, for example cryoablation and high intensity focused ultrasound (HIFU). Next to this, (focal) therapy is emerging as a treatment option for low- to intermediate-risk PCa. This therapy aims to achieve cancer cure by treating the PCa foci selectively, while preserving the rest of the prostatic gland, and thus providing a middle option between AS and radical therapy. Focal therapy (FT) is based on the ‘index lesion theory’; the largest and usually highest grade lesion indicates tumor progression and thus the prognosis [12]. Thus, the index lesion is the most clinically significant and aggressive tumor focus within the prostate and drives the natural history of the disease. Due to the multifocal nature of PCa, characterizing the index lesion is of real importance in FT when compared to whole gland treatments. In this case, targeted destruction of the index lesion should achieve an oncological effect that is equivalent to whole gland treatment. During this process of PCa detection and biopsies, imaging plays a critical role. Due to growing demand for additional minimal-invasive treatments, several techniques incorporating MR imaging have been developed.

Current standard practices for diagnosis and follow-up of suspected (recurrent) PCa include imaging by means of transrectal ultrasound (TRUS), magnetic resonance imaging (MRI) and positron emission tomography (PET)-CT. The prostate-specific membrane antigen (PSMA) PET/CT is used as a biomarker for evaluating the response to therapy and is used for the initial diagnosis and restaging of PCa. The development of the multiparametric MRI (mpMRI) was of great importance for PCa localization and volume assessment [13]. MpMRI also provides several advantages in real-time guided therapies in PCa as it allows for accurate probe placement into the lesion. In addition, MR thermometry can be used as a non-invasive real time monitoring tool in thermal ablation. [14,15]. Finally, in-bore MRI offers the opportunity to evaluate the ablation size and completeness of the ablation.

In this review, we present an overview of the current practices and challenges of the critical role held by MR imaging in the treatment of native and recurrent PCa. We will specifically focus on the role of MRI in the real time guiding of (focal) therapy.

## 2. The Role of MRI

For PCa, MR imaging plays a role in tumor detection but also in the localization, tumor volume assessment, biopsy guidance, real-time monitoring during minimal-invasive treatments and follow-up during AS of after PCa treatment. The development of mpMRI contributed to an enormous breakthrough in enabling the use of image-guided (focal) therapy in standard clinical practice [16]. Several international collaborative consensus project panels recommend performing mpMRI during the initial planning and follow-up of FT following the diagnosis of PCa based on standard biopsy [17,18,19,20].

### 2.1. Detection, Localization and Staging

**Figure 1:**
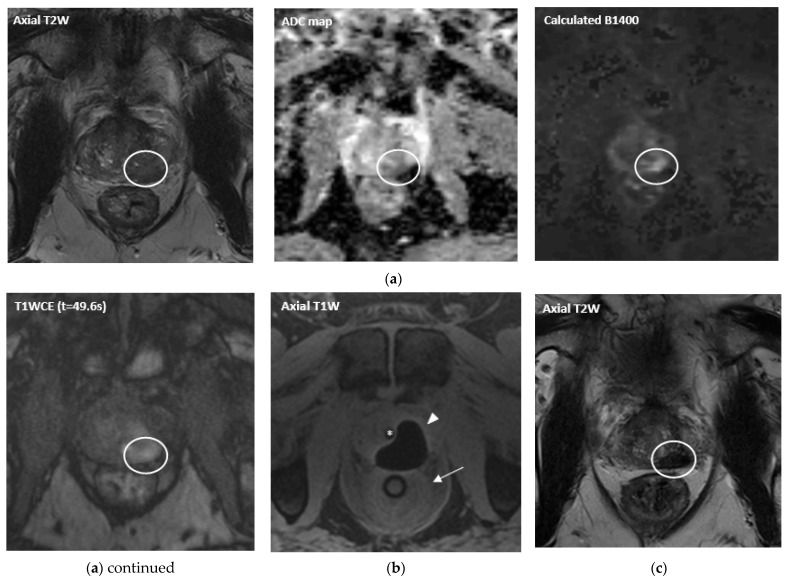

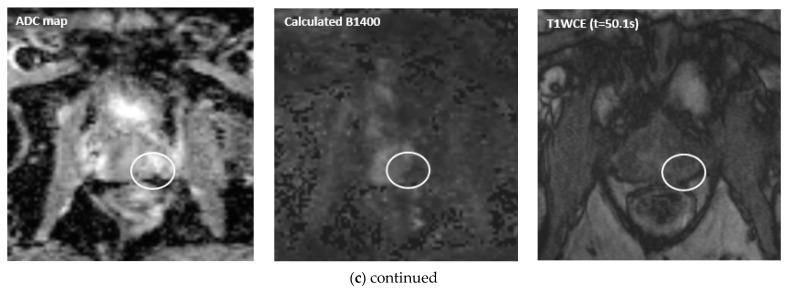
Recurrent PCa in a 72-year-old patient with a PSA level of 8.0 ng/mL (Gleason score 3 + 4 = 7) after initial treatment with external beam radiation therapy. (**a**) Pre-treatment multiparametric MR images: Axial T2W image, ADC map, high *b* value and contrast-enhanced image, respectively. Tumor is located in the left peripheral zone. The maximum lesion dimensions in anterior-posterior (AP), left-right (LR) and craniocaudal (CC) are 22, 10 and 20 mm. (**b**) Axial T1W MR image demonstrating prostate cryoablation with 3 cryoneedles (Ice-Seeds) positioned under real-time guidance. Axial image with the iceball sharply visualized as a signal void area surrounded by a hyperintense rim corresponding to 0 °C (arrowhead). The urethra (*) and rectum (arrow) contain an endoluminal urethral warming catheter and rectal warming balloon to protect the urethra and rectum from freezing, respectively. (**c**) Post-treatment multiparametric MR images at one year follow-up (PSA level of 2.2 ng/mL): Axial T2W image demonstrates the ablation zone with tissue changes. Axial ADC map and high *b* value image show no restricted diffusion. Axial dynamic contrast-enhanced MR image demonstrates a slight enhancement in the treated area. MRI-guided targeted biopsies showed a focus of adenocarcinoma with a maximum length of 1 mm. The patient continued on active surveillance.

**Figure 2:**
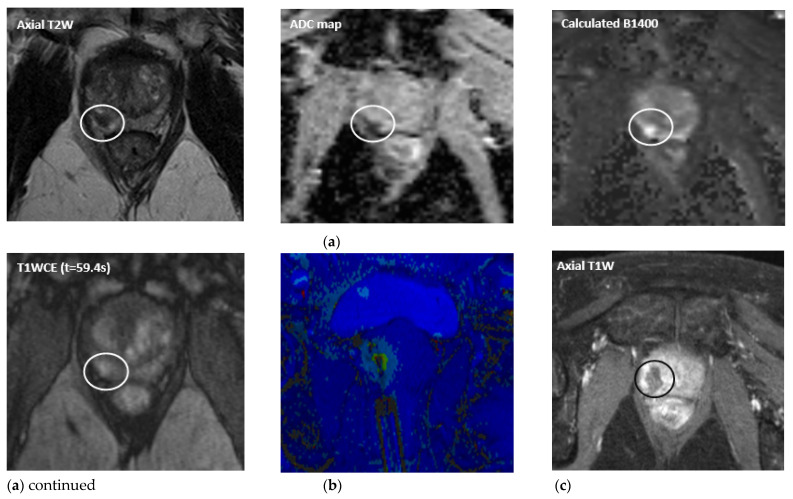

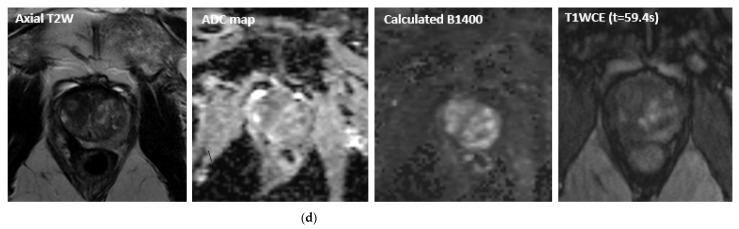
A 67-year-old patient with a PSA level of 4.6 ng/mL and Gleason 3 + 4 = 7 cancer in the right peripheral zone (PI-RADS 4). The lesion size is 7 mm in diameter. (**a**) Pre-treatment multiparametric MR images: Axial T2W image, ADC map, high *b* value and contrast-enhanced image, respectively. (**b**) Axial image of MR temperature mapping acquired during focal laser ablation (FLA). (**c**) Axial dynamic contrast-enhanced MR image acquired immediately after FLA demonstrating the non-enhancing ablation zone. (**d**) Post-treatment multiparametric MR images at one year follow-up (PSA level of 2.2 ng/mL): Axial T2W image, ADC map, high *b* value and contrast-enhanced image, respectively. There is no evidence of residual disease or recurrence.

**Figure 3:**
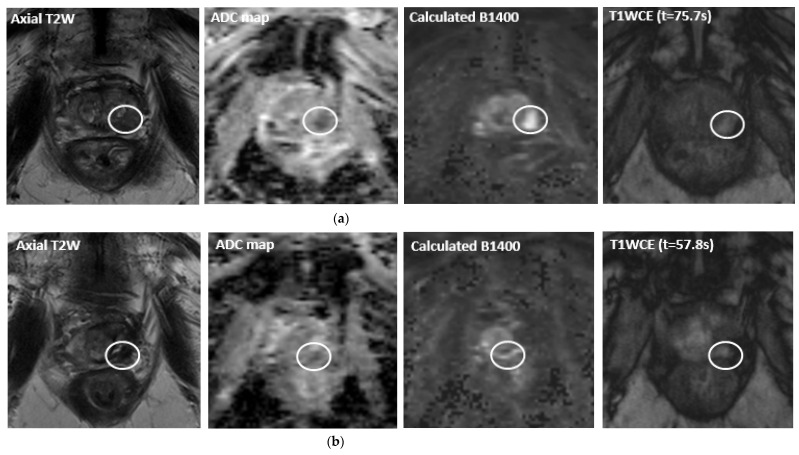
A 81-year-old patient with a positive family history of PCa, a PSA level of 5.7 ng/mL and a histopathologically proven Gleason 4 + 5 = 9 cancer in the left peripheral zone (PI-RADS 4). The maximum lesion dimensions in AP and LR are 12 and 6 mm. (**a**) Pre-treatment multiparametric MR images: Axial T2W image, ADC map, high *b* value and contrast-enhanced image, respectively. (**b**) Post-HIFU (high intensity focused ultrasound) multiparametric MR images at two years follow-up (PSA level of 6.0 ng/mL): Axial T2W image, ADC map, high *b* value and contrast-enhanced image, respectively. Residual disease is located in the left peripheral zone. MRI-guided targeted prostate biopsies proved a Gleason 4 + 4 = 8 prostate cancer.

**Figure 4:**
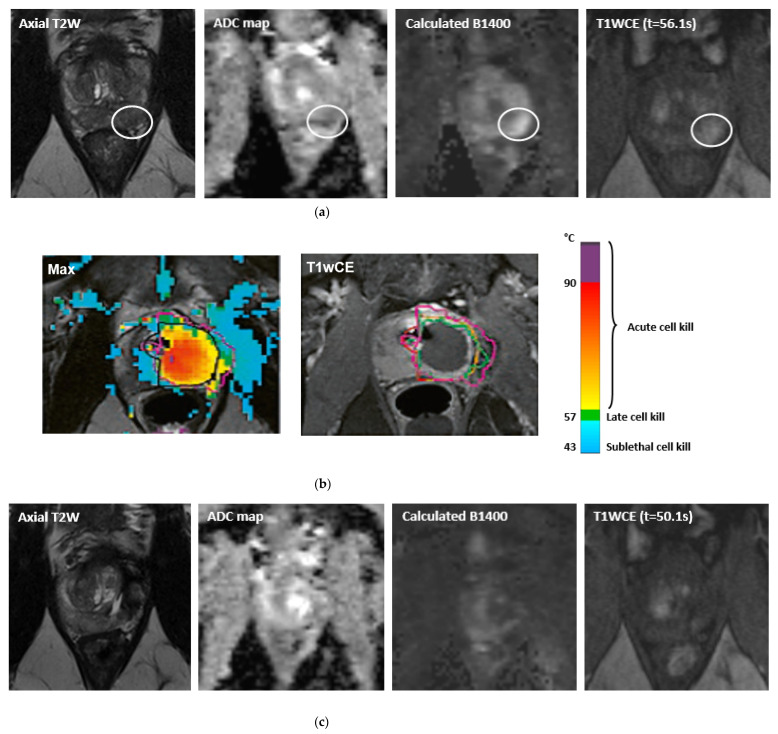
A 60-year-old patient with a PSA level of 15 ng/mL and a Gleason 3 + 4 = 7 prostate cancer in the left peripheral zone (PI-RADS 4). The maximum lesion dimensions in AP, LR and CC are 43, 46 and 35 mm. (**a**) Pre-treatment multiparametric MR images: Axial T2W image, high *b* value and contrast-enhanced image, respectively. (**b**) MR images demonstrating hemiablation on the left with transurethral ultrasound ablation (TULSA). (**c**) Post-treatment multiparametric MR images at two year follow-up (PSA level of 3.1 ng/mL): Axial T2W image, ADC map, high *b* value and contrast-enhanced image, respectively. There is no evidence of residual disease or recurrence.

MpMRI has become important with regards to noninvasive diagnostics for PCa (Figure 1a, Figure 2a, Figure 3a and Figure 4a). The mpMRI protocol of the prostate combines: (1) high-resolution anatomical T2-weighted (T2W) images performed in the axial, coronal and sagittal planes for assessing extraglandular disease with (2) functional images (multiparametric approach) performed in the axial plane, such as diffusion-weighted images (DWI) and its derivative apparent-diffusion coefficient (ADC) maps and the T1-weighted (T1W) dynamic contrast enhanced MRI (DCE-MRI). Moreover, 1.5 and 3.0 Tesla field strengths are available to perform mpMRI, with the latter having an improved spatial and temporal resolution with improved signal-to-noise ratio (SNR) [13]. The Prostate Imaging Reporting and Data System (PI-RADS) assessment uses a five-point scale which is based on the probability that a combination of the mpMRI findings correlate with the presence of a PCa lesion [13].

MpMRI is characterized by a range of high pooled sensitivity (78–91%), but relatively low pooled specificity (37–79%) for the detection of PCa [21,22,23]. As is reported by four prominent prospective multicenter studies, performing an mpMRI in patients with suspicion of PCa decreases the number of biopsy cores, reduces overdiagnosis of clinically insignificant PCa tumors, characterized by a moderately differentiated (Gleason score ≤ 6) or low volume (<0.5 mL) PCa, and detects similar rates of clinically significant prostate cancer (csPCa) as systematic biopsy in biopsy-naïve men [24,25,26,27]. Therefore, the European Association of Urology (EAU) guidelines recommend performing an mpMRI before taking prostate biopsies as a first step in the diagnostic pathway in patients with a substantial risk of having a csPCa [28].

The DCE-MRI, as part of the mpMRI, comes with several limitations, being a time-consuming and costly scan. It also depends on the use of a gadolinium-based contrast agents, requiring intravenous access and coming with the potential risk of nephrogenic systemic fibrosis [29]. To overcome these limitations, a biparametric MRI (bpMRI) protocol, which only combines T2W and DWI sequences, can be used for tumor detection. Moreover, bpMRI and mpMRI showed similar sensitivities and specificities for the detection rates [30,31,32]. Another reason to use the bpMRI for detection of PCa is the fact that DCE-MRI only plays a minor role in determining the PI-RADS Assessment Category, as it does not contribute to the overall assessment when the lesion has a low PI-RADS (1 or 2) or high PI-RADS score (4 or 5). Therefore, mpMRI may be reserved for the localization and tumor volume assessment of the lesion in patients with a high risk of having csPCa and warrant a biopsy. MpMRI, in addition to the conventional T2W images, results in more accurate PCa localization [33,34], in particular, for lesion within the peripheral zone of the prostate [35]. Furthermore, tumor volume measurement obtained by mpMRI show similar accuracy scores when compared to the reference volume measurement obtained after RP [36]. The accuracy is higher for tumors of greater volume, higher grade and when an endorectal coil was used. Although current evidence suggests that mpMRI may underestimate tumor volume, it provides necessary tumor volume information required for selecting patient for FT [37]. The potential risk for underestimation of the tumor volume should be taken into consideration and an adequate treatment margin should be preserved to ensure sufficient tumor coverage.

In the local staging process, high spatial resolution T2W imaging eventually combined with abnormal contrast enhancement on DCE-MRI, is useful in defining whether the tumor extends through the prostatic capsule, including extracapsular extension, seminal vesicle invasion and neurovascular bundle (≥T3 disease) [38].

### 2.2. Targeting

Prostate biopsy is the key element of PCa diagnosis as it enables definitive diagnosis and histopathological grading. The conventional method of acquiring prostatic tissue samples was to pursue a systematic 10–12 core biopsy of the prostatic gland using TRUS. However, systematic biopsy proved to be a less accurate tool for identifying PCa when localized in the anterior part of the gland and the distal prostatic apex [39,40]. Therefore, targeted biopsy (TB) guided by MR imaging gained more interest throughout the years along with the advances of mpMRI. According to the EAU guidelines, TB should be combined with systematic biopsy when a lesion with a PI-RADS score ≥3 is seen on mpMRI in biopsy naïve men [28] as TB results in a significantly higher diagnosis rate of csPCa and high grade PCa when compared to systematic biopsy [41].

MRI findings can be used to guide TB through three approaches: (1) TRUS-guided prostate biopsy with cognitive fusion of the MR images, (2) MRI-TRUS fusion guidance by using software that fuses the stored MR images with ultrasound by digital overlay and (3) direct in-bore guidance which is performed in the MRI suite using real-time MRI guidance. Based on the current evidence, there is no significant advantage in any of aforementioned techniques regarding the accuracy in the detection of csPCa [42,43].

### 2.3. Real-Time Treatment Monitoring and MR Temperature Mapping

The extent to which MRI can be used is not limited to detection only, but it is also useful for localization and targeting of the tumor with biopsies. Moreover, MR imaging is used to monitor the treatment in real-time. Cryoneedles and laserfibers, used in cryoablation and focal laser ablation (FLA), respectively, can be inserted into the tumor under real-time image guidance and treatment effects can be visualized in multiple planes through real-time treatment monitoring. Moreover, temperature mapping is used for real-time treatment monitoring in thermal ablation therapies. During these therapies, i.e., FLA, HIFU and transurethral ultrasound ablation (TULSA), MR thermometry can be used to provide real-time temperature feedback to ensure accurate tumor ablation and the preservation of the surrounding healthy tissues (Figure 2b and Figure 4b).

There are several methods in which noninvasive temperature mapping with MRI is possible: (1) proton density, (2) T1 and T2 relaxation time of water protons, (3) diffusion coefficient (D), (4) magnetization transfer, (5) temperature-sensitive contrast agents and (6) proton resonance frequency (PRF) shift of water protons [44]. PRF MR thermometry is the most frequently used method for measuring temperature-related changes and is based on the fact that water hydrogen bonds will disrupt at elevated temperatures. This results in an increased shielding constant of the protons, decreased chemical shift and thereby decreased resonance frequency for water protons [45]. The screening constant will increase in a linear fashion as the temperature increases. The gradient-recalled echo (GRE) sequence is used to acquire phase maps during the procedure (during heating) and baseline phase maps (preheating). The relative phase shift is then determined by calculating the difference between these phase maps. The temperature maps are created by a software that converts this relative temperature difference in color maps that are then overlaid in the anatomical T2W images. Additionally, a ‘thermal dose’ model, based on the Arrhenius model, can be used to relate the treatment temperature to the subsequent dose estimations that lead to thermal tissue damage.

During cryoablation the temperature decreases below the freezing point. Because the majority of the MR signal comes from the protons in the hydrogen nuclei of water molecules, MR thermometry is not impractical in cryoablation, given that strong hydrogen bonding occurs between water molecules at low temperatures. This free water shifts into ice crystals and does not contribute to the MR signal. Ice is therefore represented as a black signal void. However, several studies have demonstrated that MR thermometry can be feasible in cryoablation by using ultrashort echo times (UTE) imaging [46,47,48]. The theory behind this technique is that a fraction of the tissue water in frozen tissue remains mobile, and, therefore, is the source of the MR signal in UTE. However, this technique has not been proved to be clinically applicable due to the fact that (near) real-time imaging is not possible. Even though the relative tissue temperature cannot be measured during cryoablation, the treatment can be monitored in (near) real-time by imaging the iceball which is depicted as a sharply delineated signal void. In fast T1W GRE images, a thin rim of increased signal is visible around the iceball (Figure 1b). This bright hyperintense rim surrounding the iceball corresponds to cooled but nonfreezing temperatures (<20 °C) proximal to the frozen zone [49]. Thus, the monitoring of this hyperintense rim could be used to maintain a safe margin from vital adjacent structures during cryoablation. Additional temperature monitoring during cryoablation can be achieved by MR compatible invasive thermocouple probes.

## 3. The Application of MRI in Real-Time Guided Therapy

Once the lesion is identified within the prostate gland by imaging and biopsy, further steps can be taken towards personalized treatment. There are several methods available for performing MRI-guided therapies in both patients with native and recurrent PCa with each their own pros and cons (Table 1), of which we will discuss cryoablation, FLA, HIFU and TULSA in this review.

### 3.1. Cryoablation

Cryotherapy was initially used for whole gland ablation [50] and has been adapted for FT. Cryoablation uses often two, and sometimes three, freeze-thaw cycles and is usually performed under general anesthesia. Several cryoneedles are transperineally placed in the prostate under MR-image guidance (Figure 1b). Before the first freezing cycle is initiated, a urethral warming catheter is inserted to allow for a warmer fluid (±43 °C) to be circulated as to prevent urethral freezing and sloughing. [51]. If indicated, a rectal warming balloon is inserted in order to reduce the risk of rectal fistula forming. Then fast freezing is induced with temperatures preferably below −40 °C followed by slow thawing which causes irreversible cell necrosis and apoptosis. The created area of ablation during freezing is monitored and is recognized as an ‘iceball’. MRI-guided cryoablation allows real time monitoring of the iceball growth, and, thus, ensures that a safe distance to the surrounding vital structures, urethra or rectum, is acquired [52,53].

Cryoablation can be performed in both newly diagnosed as in recurrent PCa. In patients with native PCa, two studies focused on MRI-guided cryoablation as a whole gland therapy with a smaller number of patients (4 patients) [54] and another cohort including 30 patients [55] in patients with native PCa. These studies showed that whole gland cryoablation may prove the answer in patients in whom standard treatment, i.e., RP of radiotherapy, may be complicated due to prior pelvic surgery and/or radiation due to other malignancies.

Cryoablation is also performed as a salvage treatment in patients with recurrent PCa. The first feasibility study in 11 patients by Gangi et al. in 2011 showed promising results regarding whole gland cryoablation under MR guidance and opened the door to further research [56]. Two recent studies have looked retrospectively at the outcomes of focal MRI-guided cryoablation in a salvage setting [57,58]. Overduin et al. assessed salvage prostate cryoablation in 47 patients with biopsy proven local recurrence after primary radiotherapy. The authors suggested that a minimum of 5 mm iceball margin around the tumor is required in order to achieve sufficient cryoablation and a higher local progression-free survival. More recently, the study of Bomers et al. investigated the quality of life (QoL) and the disease-free survival in 62 patients with radiorecurrent PCa who underwent MRI-guided cryoablation with a follow-up of ≥12 months. Although the participants showed increased symptoms of incontinence and declined erectile function after 12 months, this did not affect their QoL. With this preservation of QoL and local PCa control, with a disease-free rate of 83% and 63% at 6- and 12-months, respectively, the authors concluded cryoablation to be a reasonable alternative to salvage RP.

Other investigators have focused on techniques that may prevent rectal injury for patients who undergo MRI-guided cryoablation, such as rectal wall displacement with saline [59] and transperineal injection of autologous blood into the interprostatorectal space [60]. More research is needed in order to establish a consensus as to whether these alternatives could provide more protection than a rectal warming balloon.

### 3.2. Focal Laser Ablation

FLA, also known as laser-induced thermal interstitial therapy or interstitial photothermal therapy, is a technique that is based on tumor ablation through hyperthermia. This causes interstitial damage and coagulative necrosis. The procedure can be performed using the transperineal, transrectal or transgluteal approach. Guided by mpMRI, titanium trocars and guide catheters are inserted to the targeted depth confirmed with the MR images before activating the laser. A single ablation takes around 1–2 min with a minimum target temperature of 60 °C. MR thermometry is used for monitoring appropriate heating and cooling of the prostate and adjacent structures (Figure 2b) [61]. The Arrhenius formula is used to calculate thermal damage [62]. Temperature and ablation maps are overlaid in the anatomical T2W images. Immediate post-ablation T1W DCE-MRI is performed to confirm devascularization of the target as is demonstrated by the absence of enhancement (Figure 2c) [63,64]. Before the ablation, a (temporarily) urethral Foley catheter might be placed to prevent acute urinary retention after the treatment due to edema of the tissue around the ablation zone. The advantage in FLA above other focal treatments is the ability to perform it as an outpatient procedure under regional anesthesia (periprostatic nerve block) or conscious sedation.

Several feasibility and phase I prospective studies have shown MRI-guided FLA to be a feasible and safe FT option for patients with localized low- to intermediate-risk PCa without significant complications [65,66,67,68].

The phase II study of Eggener et al. in 27 patients demonstrated a potential cure with good functional outcomes for continence and potency with a follow-up period of 12 months [69]. Another small-scale longitudinal outcome study showed excellent early oncological and functional outcomes in 25 patients at a 3-months follow-up while also confirming the feasibility and safety of MRI-guided FLA [70]. Three more studies have investigated the oncological and functional benefits of this treatment in patients with low- to intermediate-risk PCa with a follow-up period beyond one year [71,72,73]. The study of Chao et al. showed a failure-free survival of 83% with a median follow-up of 71 months [71]. The recurrence rate of in-field, out-of-field and the combination of in- and out-of-field PCa was 36%, 4% and 4%, respectively. Walser et al. investigated the oncological outcome in a larger cohort of 120 patients with a median follow-up time of 34 months [72]. Posttreatment, 44 patients (36%) had positive MR imaging (PI-RADS ≥ 3) or persisting PSA elevation. Prostate biopsy confirmed clinically insignificant PCa tumors (Gleason score ≤ 6) in 4 patients (9%) and csPCa (Gleason score ≥ 7) in 18 patients (41%). Moreover, the one-year retreatment-free rate was 83% with only 20 patients (17%) requiring secondary therapies, including reablation. In the study of Hakeem et al., treatment success, defined as the absence csPCa in ablated areas based on TB and systematic biopsy, was achieved in 80% of the patients, showing encouraging oncologic control [73]. Moreover, the scores for functional outcome did not show a significant statistical decrease compared to baseline during follow-up.

Finally, one recent study investigated the possibility of FLA in recurrent PCa. The authors demonstrated MRI-guided FLA to be a feasible salvage treatment after MRI-guided HIFU recurrence [74].

### 3.3. High Intensity Focused Ultrasound

The MRI-guided HIFU device consists of an endorectal transducer without the need for transperineal or transrectal needle placement. High frequency ultrasound waves are generated by an endorectal transducer and focused by an acoustic lens or phased array transducers to a target area [75]. Due to the high concentration of acoustic energy on a local spot, the tissue is heated rapidly at temperatures of 60–95 °C, and thus damaged by acoustic cavitation and coagulative necrosis. The transducer contains a built-in cooling system which circulates degassed water that actively cools the rectal mucosa to avoid rectal thermal damage [76]. The procedure is performed under general or spinal anesthesia. After the endorectal transducer is placed, MR imaging is performed. Then, the target area is manually or electronically contoured. The device generates a patient-specific treatment plan including the required energy level and number of sonications, and specific targeting is confirmed by MR thermometry [77]. A Foley catheter is placed for 1–2 weeks posttreatment in order to avoid urinary retention due to edema caused by the thermal effects.

HIFU is one of the most studied and used forms of ultrasound-guided ablation treatments, particularly when updated systems with image fusion of mpMRI with ultrasound systems became available, thereby increasing the accuracy of lesion localization [78,79].

At present, the ExAblate prostate system (InSightec Inc., Haifa, Israel) is the only device available for MRI-guided HIFU in PCa treatment. The first clinical results were published in 2012 with the study of Napoli et al. showing accurate coagulative necrosis in the treatment zone with no residual tumor in the ablated area in the histopathology after RP [77]. Along with several other small cohort preliminary studies [80,81,82], it demonstrated MRI-guided HIFU to be a feasible and safe therapy with promising initial post-therapy outcomes.

As yet, two phase I [83,84] and one phase II [85] clinical trials have been performed with MRI-guided HIFU. A prospective study enrolled a cohort including 8 patients who were treated for their low- or intermediate-risk PCa at 10 target sites [83]. Six months after treatment, a successful ablation is seen in 6 out of 10 target sites. In one patient the biopsy samples revealed a 2 mm Gleason score 4 + 4 = 8 lesion, after which the patient subsequently underwent RP with negative surgical margins. After ablation three patients were placed on AS for their Gleason score 6 disease, two of which were diagnosed with a Gleason score 3 + 4 = 7 disease prior to treatment. No changes in QoL parameters were seen between baseline and 6 months in 6 out of 8 patients. Tay et al. performed a larger phase I study with a two-year follow-up in 14 patients [84]. The procedure was well tolerated by all patients. In 7 patients, grade 1–2 complications in the Clavien–Dindo classification were seen, including one urinary tract infection, one epididymo-orchitis, both resolved with appropriate antibiotics and five self-limiting hematuria. At 6- and 24-month transperineal template biopsy, one and three men had a Gleason score of >6, respectively. The phase II clinical trial of Ghai et al. published in 2021, showed promising oncologic and functional outcomes in 44 patients [85]. After 5-months of follow-up, 41 patients (93%) were free of csPCa. One patient with residual disease was successfully treated with MRI-guided FLA and the two remaining patients remained on AS. Moreover, median International Index of Erectile Function-15 (IIEF-15) and International Prostate Symptom Score (IPSS) scores were similar at baseline and at 5 months.

### 3.4. Transurethral Ultrasound Ablation

MRI-guided transurethral ultrasound ablation (TULSA) is a novel ablation technique that serves as an alternative transurethral approach compared to the transrectally performed HIFU. Although initially used for whole gland ablation, some studies have investigated TULSA for FT and partial gland treatment [86,87,88,89]. The device currently used for MRI-guided TULSA is the TULSA-PRO System (Profound Medical Inc., Toronto, ON, Canada). An ultrasound applicator (UA) with a linear array of 10 independent ultrasound transducer elements is inserted in the prostatic urethra [90]. This heating applicator delivers high-intensity directional (but unfocused) ultrasound energy to the adjacent prostatic gland, thereby causing thermal damage. Degassed water (10–40 °C) is circulated through the UA in order to cool and preserve 1–2 mm of urethral tissue [91]. An endorectal cooling device (ECD), which circulates water at a constant desired temperature, is inserted to prevent rectal tissue damage. TULSA is typically performed completely within the MRI suite and under general anesthesia. High resolution images are displayed for device positioning and treatment planning and temperature maps are used for monitoring and controlling the treatment by adjusting the intensity and frequency of ultrasound beams and the rotation rate of the UA (Figure 4b). The software controls the temperature at the rims of the ablation zone through a real time feedback loop, thereby maintaining a constant temperature of approximately 57 °C for the ablation zone in order to preserve the neurovascular bundles [92].

Studies that enrolled patients with localized PCa for undergoing TULSA prior to RP, proved TULSA to have excellent spatial targeting accuracy when compared to histologically proven PCa lesions after RP [86,93]. The single-center phase I study of Anttinen et al. enrolled 6 patients who underwent RP, as the reference standard, three weeks after MRI-guided TULSA [88]. Ablation accuracy of 1.7 ± 0.4 mm have been observed. The histopathology after RP showed no viable cancer within the ablated zone. Another prospective phase I clinical trial investigated the safety and feasibility of MRI-guided TULSA for whole gland ablation in 30 patients at three tertiary referral centers with a 12-months follow-up [94]. No intraoperative complications were seen and after treatment there were no severe urinary incontinence, rectal injury or fistula. This experience was recently updated in 22 patient who completed the 3-year follow-up in this trial [95]. Urinary, bowel and erectile function remained sTable 3 years after TULSA. The salvage-free survival rate was 76% at 3 years follow-up.

The TACT study, to date the largest multicenter trial within 115 men across 13 centers, demonstrated effective and accurate tissue ablation in whole gland TULSA (0.1 ± 1.4 mm measured on MR thermometry) accompanied with low rates of toxicity and residual disease [96]. Grade 3 (severe) adverse events occurred in 9 patients (8%), and there were no Grade 4 events. A PSA reduction of ≥75% was achieved in 110 men (96%). At 12-month biopsies 72 out of 111 patients (65%) had no evidence of cancer and 16 patients (14%) had low volume grade group 1 PCa. Moreover, a potency rate of 75% (69 out of 92 men) was seen by 12 months.

Other studies have explored the possibility for TULSA to serve as either a whole or partial gland salvage treatment in localized radio recurrent PCa [89] and as a palliative treatment in patients with symptomatic locally advanced PCa as opposed to the current gold standard of palliative transurethral resection of the prostate [97]. Although they show promising results, more research is needed in order to be able to definitive conclusions.

## 4. Clinical Follow-Up

Although PSA can be used as a non-imaging biomarker during follow-up after performing FT, the PSA value alone is insufficient to determine oncological success due to the fact that, unlike after radical treatment, the non-treated part of the prostate gland is still able to produce PSA. However, although the significance of PSA is diminished after FT, the relative decrease of PSA before and after FT plays a role in predicting the success of ablation of the index lesion [18].

In addition to PSA, MR imaging is an important diagnostic tool in the follow-up after MRI-guided (focal) treatment. Particularly, mpMRI is the state of the art imaging modality for follow-up after FT [18]. As part of the mpMRI protocol, mainly DCE-MRI is a powerful tool regarding the follow-up after (focal) PCa treatment, as it increases the sensitivity of recurrence detection in patients who have been treated with EBRT [98,99,100], as well as it increases the diagnostic accuracy, sensitivity and specificity of recurrence detection after RP [100,101,102,103]. Several changes may be present after FT, such as loss of zonal differentiation, thickening of the prostatic capsule, periprostatic fibrosis and scarring, depending on the extent and type of treatment [18]. Occasionally, features that are typically associated with recurrence, i.e., a hypointense T2 signal or the combination of hyperintense signal on high *b* values (≥b1400) DWI with hypointense signal on ADC map, may not represent recurrence in the ablated area. Early soft tissue contrast enhancement on T1W images is therefore needed in order to specify whether there is a suggestion of residual disease or recurrence (Figure 1c, Figure 2d, Figure 3b and Figure 4c). Initial follow-up mpMRI should take place 6 months and 1 year post treatment [18,19]. After the first year, it is advised to have annual follow-up with mpMRI for the next 5 years following treatment [19].

Further follow-up assessment should consist of the combination of MRI-targeted biopsies, either MRI-TRUS fusion or direct in-bore biopsies, and systematic 12-core biopsy to evaluate the treated and untreated area, respectively [17,19,20]. The first biopsy should be obtained one year after initial treatment, and thereafter only when there is a suspicious lesion on imaging, a rise in PSA and/or an abnormal digital rectal examination (DRE) [17,19,20]. Finally, PSA should be taken every 3 months within the first year after treatment, and thereafter every 6 months for a minimum of 5 years [19,20].

## 5. Discussion and Conclusions

Prostate MRI enabled the translation of biopsy targeting and MRI real time guided (focal) treatment in patients with localized (recurrent) PCa, which has led to a widespread use in clinical practice. Advantages of excellent detection, localization and targeting, combined with the ability of real-time monitoring and temperature mapping, make MRI-guided interventions suitable for targeted treatment with acceptable functional and oncological outcomes.

Although MRI-guided interventions offer several advantages, some technical challenges and limitations still remain. Since MRI is receiving a more prominent role and is used in both detection and targeting with biopsies, it stands as a challenge to keep costs low when also used to guide interventions. Moreover, the costs will also increase due to the need for MRI compatible materials as well as MRI scan times. Another disadvantage is the limited space within the MRI bore, thereby limiting the range of motion of the physicians in the magnet.

Despite the fact that ultrasound guidance offers a practical, low-cost, real-time imaging modality, the visualization of surrounding vital structures is limited during the delivery of ablative energies. Disadvantages of performing ablative treatments under ultrasound guidance is the lack of providing accurate PCa localization (although fusion can be used) and the inability to monitor the temperature during the procedure. With regard to cryoablation, another drawback is the acoustic shadowing artifact from the posterior rim of the ice ball. Conversely, MRI-guided cryoablation allows visualization of the iceball in multiple planes which allows more precise cryoprobe placement and iceball monitoring during the treatment.

MRI-ultrasound fusion has been suggested as a third imaging modality for real-time monitoring during PCa interventions. Several studies have investigated MRI-ultrasound fusion in FLA [104,105], cryoablation [106] and HIFU [78,79,107,108,109]. Advantages to this procedure are its cost-effectiveness as compared to MRI-guided procedures without ultrasound fusion and the ability to perform the procedure in a clinical, out of bore, setting. Unfortunately, MRI-ultrasound fusion (focal) therapy lacks the high spatial resolution of MRI, the option of protecting the rectal wall with either a rectal cooling (during TULSA) or heating device (during cryoablation) to prevent it from damage and the possibility for temperature mapping. To overcome the latter issues, interstitial thermal probes, thermocouples [110,111] or contrast enhanced ultrasound [112] are used for temperature monitoring resulting in an excellent measure of the actual treatment effect and no injury to the rectal wall, the prostate or the pelvic organs.

New future technologies may provide a promising perspective for upcoming MRI-guided PCa therapies. In order to keep costs low, it is crucial to ensure the procedure time remains low in addition to reducing the amount of comorbidities compared to standard of care. An important measure to accelerate the treatment is the use of MRI compatible robots to guide both the biopsy and treatment [113,114,115]. Furthermore, new advances in the use of artificial intelligence and deep learning for lesion classification and lesion detection in PCa extracted from T2W sequences and DWI with ADC maps [116] and new MR sequences could improve PCa diagnosis by improving the imaging quality [117,118] and accelerate the acquisition time [119,120,121]. Moreover, an appropriate patient selection is needed. This relies on improved accuracy in diagnosis as well as tumor delineation, thereby improving the eradication of the lesion. Improvement in diffusion weighted imaging resolution may help to achieve this. Moreover, low field MRI in combination with artificial intelligence may be an opportunity to provide a more accurate diagnosis. Finally, future studies regarding the follow-up after ablation are needed in order to lead to improved consistency in definitions, outcome reports and guidelines in frequency and type of follow-up.

## Figures and Tables

**Table 1:** Summary of pros and cons of (focal) MRI-guided therapies in PCa.

Ablation Modality	Mechanism of Activity	Pros and Cons
Cryoablation	Cryoablation induces irreversible localized cell destruction by freezing followed by thawing	Cons: Real-time temperature mapping is not applicable Smaller lesions are more difficult to treat
FLA	FLA causes photothermal injury which leads to coagulative necrosis	Pros: Possibility to perform using the transperineal, transrectal or transgluteal approach Ability to perform under local anesthesia in an outpatient setting Real-time temperature mapping Cons: Only feasible for small lesions
HIFU	HIFU uses ultrasound energy focused by an acoustic lens to cause tissue damage	Pros: Real-time temperature mapping Cons: May not be suitable in patients with prostate calcifications Less suitable for ventral lesions
TULSA	TULSA delivers high-intensity directional (but unfocused) ultrasound energy which causes thermal damage	Pros: Ability to perform focal-, hemi- or whole gland ablation Real-time temperature mapping Cons: May not be suitable in patients with prostate calcifications

## Data Availability

Not applicable.
